# Dendritic Cell-Mediated, DNA-Based Vaccination against Hepatitis C Induces the Multi-Epitope-Specific Response of Humanized, HLA Transgenic Mice

**DOI:** 10.1371/journal.pone.0104606

**Published:** 2014-08-11

**Authors:** Sasmita Mishra, Bianca J. Lavelle, Joe Desrosiers, Matt T. Ardito, Frances Terry, William D. Martin, Anne S. De Groot, Stephen H. Gregory

**Affiliations:** 1 Department of Medicine, Rhode Island Hospital and the Warren Alpert Medical School of Brown University, Providence, Rhode Island, United States of America; 2 Institute for Immunology and Informatics, University of Rhode Island, Providence, Rhode Island, United States of America; 3 EpiVax, Inc., Providence, Rhode Island, United States of America; KAIST, Graduate School of Medical Science & Engineering, Republic of Korea

## Abstract

Hepatitis C virus (HCV) is the etiologic agent of chronic liver disease, hepatitis C. Spontaneous resolution of viral infection is associated with vigorous HLA class I- and class II-restricted T cell responses to multiple viral epitopes. Unfortunately, only 20% of patients clear infection spontaneously, most develop chronic disease and require therapy. The response to chemotherapy varies, however; therapeutic vaccination offers an additional treatment strategy. To date, therapeutic vaccines have demonstrated only limited success. Vector-mediated vaccination with multi-epitope-expressing DNA constructs alone or in combination with chemotherapy offers an additional treatment approach. Gene sequences encoding validated HLA-A2- and HLA-DRB1-restricted epitopes were synthesized and cloned into an expression vector. Dendritic cells (DCs) derived from humanized, HLA-A2/DRB1 transgenic (donor) mice were transfected with these multi-epitope-expressing DNA constructs. Recipient HLA-A2/DRB1 mice were vaccinated s.c. with transfected DCs; control mice received non-transfected DCs. Peptide-specific IFN-γ production by splenic T cells obtained at 5 weeks post-immunization was quantified by ELISpot assay; additionally, the production of IL-4, IL-10 and TNF-α were quantified by cytokine bead array. Splenocytes derived from vaccinated HLA-A2/DRB1 transgenic mice exhibited peptide-specific cytokine production to the vast majority of the vaccine-encoded HLA class I- and class II-restricted T cell epitopes. A multi-epitope-based HCV vaccine that targets DCs offers an effective approach to inducing a broad immune response and viral clearance in chronic, HCV-infected patients.

## Introduction

Hepatitis C virus (HCV), a small single-stranded RNA virus, is a major cause of chronic liver disease. An estimated 180 million people worldwide, ∼3 million in the United States alone, are infected [1]–[3]. The vast majority of those (80–85%) fails to clear the virus spontaneously, most patients develop chronic hepatitis C [4]. Hepatitis C is the underlying cause of ∼25% cases of liver cancer (WHO Media Center Report, 2012). Cirrhosis due to chronic HCV is the leading indicator for liver transplantation in the U.S. The socioeconomic burden of hepatitis C-associated diseases in the U.S. is estimated at a staggering $7 billion/year [5]. Until recently, the standard of care for patients consisted of pegylated-interferon and ribavirin administered over a 48-week period, which results in a sustained virologic response (SVR) in 45–50% of those infected with HCV genotype 1 (the principal causative agent in the US) [6]. New therapeutic approaches that include direct-acting anti-viral agents, for example, NS3-4A protease inhibitors (i.e., boceprevir or telaprevir) and NS5B polymerase inhibitors (e.g., sofosbuvir), administered in various combinations with or without pegylated-interferon or ribavirin, increase the SVR significantly [7]. Unfortunately, the dosing regimens can be complex, the adverse side effects occur frequently and are often severe, the cost of treatment is high, and a significant portion of treated population remains infected. Consequently, it seems highly unlikely that these emerging treatment paradigms will exert an immediate impact on the global distribution of HCV. Additional treatment approaches are urgently needed.

The ability of a minority of patients to resolve acute HCV infections spontaneously, and the capacity of some individuals to eliminate virus in the absence of antibody production, suggest that an effective, epitope-based therapeutic vaccine is a realistic goal. Development of such a vaccine has proven problematic, however, due primarily to: infidelity of the viral RNA polymerase (NS5b), genetic diversity and the rapid emergence of viral variants [8]. Thus, a safe and effective vaccine must elicit the vigorous responses of both CD4^+^ and CD8^+^ T cells to conserved viral epitopes, which contribute to the elimination of HCV without causing liver pathology. Vaccine strategies undertaken to date, however, have achieved varied and only limited success in clinical trials [9], [10]. Therapeutic vaccination with HCV epitope-expressing dendritic cells (DCs) concurrent with or without conventional drug therapy offers a vector-mediated approach to treating chronic, HCV-infected patients.

DCs play a central role in CD4^+^ and CD8^+^ T cell activation and the induction of immunity [11]. The potential efficacy of DC vector-mediated vaccines in treating chronic hepatitis C was demonstrated in a recent Phase I clinical trial in which patients vaccinated with autologous, monocyte-derived DCs pulsed with 6 HCV-specific, HLA-class I-restricted peptides exhibited peptide-specific CD8 T cell responses [12], [13]. These responses were not sustained, however, and the effect on viral load was negligible suggesting that HCV clearance requires vaccination with a broader range of epitopes that includes those restricted by HLA class II [12]. Toward this end, immunoinformatics tools were used to predict 19 HLA-A*0201-restricted epitopes and 19 promiscuous, HLA-DRB1-restricted immunogenic consensus sequences (ICS, each composed of 5–28 epitopes), which were highly-conserved among HCV genotypes 1a and 1b [14]. These predicted epitopes/ICS exhibited HLA binding activity in competitive binding assays and the ability to elicit the peptide-specific responses of naïve human T cell *in* *vitro*, thus validating their immunogenicity [14]. In the experiments described herein, the gene sequences that encode these peptides were incorporated into multi-epitope-expressing DNA constructs; humanized, HLA-A2/DRB1 transgenic mice were then vaccinated with these constructs using DCs as a vector. Splenocytes prepared at 35 days post-immunization exhibited peptide-specific IFN-γ and TNF-α production in response to the vast majority of the vaccine-encoded HLA class I- and class II-restricted epitopes; IL-4 and IL-10 production was negligible.

## Materials and Methods

### Multi-HCV epitope DNA vaccine constructs

Essentially the entire human population expresses one or more of a panel of the eight common HLA-DRB1 alleles: DRB1*0101, *0301, *0401, *0701, *0801, *1101, *1301, and *1501; approximately 50% expresses HLA-A*0201 [15], [16]. Accordingly, 19 HLA-A*0201-restricted epitopes and 19 promiscuous, HLA-DRB1-restricted ICS were predicted, synthesized as peptides, and validated by demonstrating their HLA binding activity and ability to elicit immune recognition by naïve human T cells *in* *vitro* [14]. These sequences are listed in Tables S1 and S2 in File S1.

The sequences encoding these A2- and DRB1-restricted epitopes/ICS were aligned in a “string-of-beads” conformation, concatenated initially at random to form two multi-epitope genes: DRB1 and A2 constructs. Epitope order was then optimized to avoid creation of immunogenic sequences at epitope junctions using VaccineCAD, an algorithm that iteratively re-arranges epitopes [17]. Where VaccineCAD re-ordering was not sufficient to reduce potential junctional immunogenicity, Ala-Ala-Tyr proteasomal cleavage (Class I) or Gly-Pro-Gly-Pro-Gly spacer (Class II) sequence was inserted between epitopes to optimize processing [18].

A Kozak sequence (GCCGCCACCATGG) was engineered upstream of the coding sequence to ensure efficient translation initiation. For the DRB1 construct, the tissue plasminogen activator secretion sequence (QMSPALTCLVLGLALVFGEGSA) was inserted between the start codon and the multi-epitope coding sequence to target the protein product to the extracellular space and the MHC class II processing pathway. For the A2 construct, a calreticulin sequence was inserted between the start codon and the multi-epitope coding sequence to target the protein product to the endoplasmic reticulum and the MHC Class I processing pathway [19]. Finally, two stop codons were inserted following the multi-epitope coding sequences to ensure efficient translation termination. The two multi-epitope gene sequences were synthesized by GenScript USA, Inc. (Piscataway, NJ) and cloned into the NTC8685-eRNA41H-EGFP expression vector (Nature Technology Corporation, Lincoln, NE).

### Mice

A colony of humanized, HLA double-transgenic mice [HLA-A2.1-/HLA-DRB1-transgenic H-2 class I/class II-knockout mice, obtained from Dr. Y-C. Lone (Paris, France) [20], is currently housed and bred under contract at Taconic Farms, Inc., Germantown, NY. Mice, housed in well-ventilated rooms maintained at 22°C and an alternating 12-hour light and dark cycle, were provided food and water ad libitum. All experiments were conducted using female animals between 6 and 8 weeks of age at the time of initiation.

### Immunization

**Figure 1:**
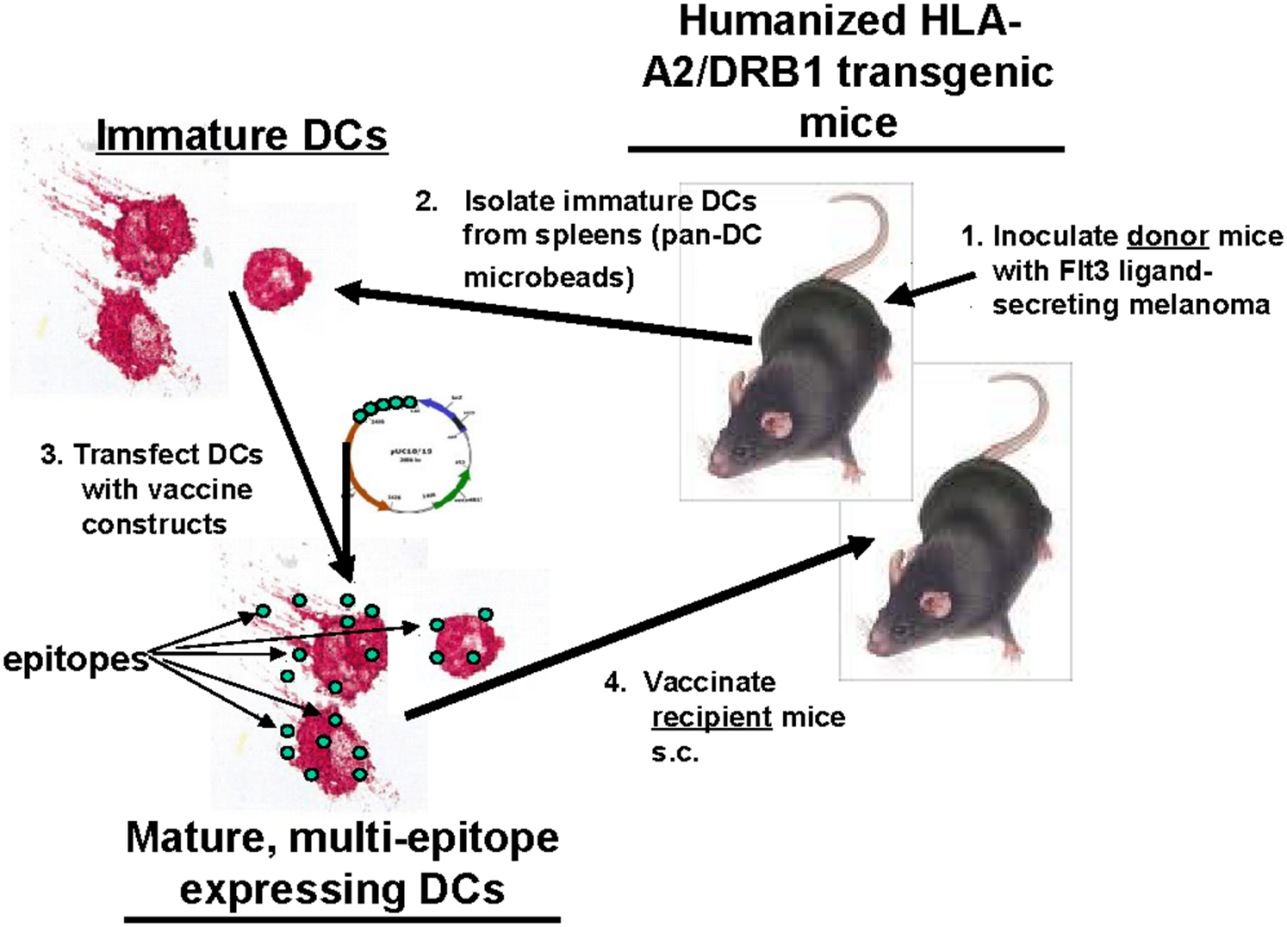
Schematic: DC vector-mediated vaccination of HLA-A2/DR1 mice. Humanized HLA-A2/DRB1 transgenic mice were vaccinated s.c. with multi-HCV epitope-expressing plasmids using DCs derived from transgenic mice administered Flt3L-secreting B16 melanoma cells as a vaccine vector.

HLA-A2/DRB1 transgenic mice were immunized with the two multi-epitope-expressing DNA constructs using DCs as a vector according to the schematic shown in Figure 1. Briefly, 2–3 donor mice were inoculated s.c. with 1×10^7^ FMS-related tyrosine kinase 3 ligand (Flt3L)-secreting B16 melanoma cells (gift obtained from Dr. Glenn Dranoff, Dana-Farber Cancer Institute, Boston, MA), which induced a dramatic expansion of the DC population *in* *vivo* [21], [22]. On day 12–14 post-inoculation, single cell splenocyte suspensions were prepared and rendered erythrocytes-depleted by ammonium chloride treatment. An enriched DC population was obtained by positive selection using pan-DC (anti-CD11c and anti-mPDCA-1) magnetic MicroBeads and the protocol provided by the supplier (Miltenyi Biotec Inc., Auburn, CA). Subsequently, these purified DCs were transfected with either the multi-epitope-expressing A2 or DRB1 vaccine construct using the mouse dendritic cell nucleofector kit, AMAXA 4D-Nucleofector System and protocol provided (Lonza, Walkersville, MD). DCs obtained from a new group of donor mice each time were transfected with these two constructs and mixed in equal numbers just prior to vaccinating recipient animals.

Groups of four recipient mice were inoculated s.c. at the base of the tail at 2-week intervals with 2×10^6^ DCs transfected with the HLA-A2 and HLA-DRB1 vaccine constructs; mice vaccinated with 2×10^6^ non-transfected dendritic cells served as the control. Spleens were dissected at 4–5 weeks post-immunization, and the peptide-specific responses of T cells derived from vaccinated and control mice were determined.

### Flow cytometric analysis

The cell-surface markers expressed by the purified CD11c^+^PDCA-1^+^ DCs were analyzed by flow cytometry using the methods we described previously [23], [24] and the following panel of dye-conjugated monoclonal antibodies purchased from BD Biosciences (San Jose, CA): CD8α (clone 53-6.7), CD11b (clone M1/70), CD11c (clone HL3); CD40 (clone MH40-3), CD45R/B220 (RA3-6B2), CD80 (clone 16-10A1), CD86 (clone GL-1) and HLA-DR (clone L243). All staining was conducted in the presence of saturating concentrations of anti-CD16/CD32 (Fcγ III/II receptor-block, clone 2.4G2); appropriate rat, hamster, and mouse IgG isotype matched controls were included in the analyses to correct for non-specific staining. Flow cytometric analyses were performed using a Becton Dickinson LSR-II flow cytometer (BD Biosciences, San Jose, California) and analyzed using FlowJo software (Tree Star, Inc., Ashland, OR).

### ELISpot assays

Epitope-specific T lymphocytes were quantified using mouse IFN-γ ELISpot assay kits purchased from eBioscience, Inc. (San Diego, CA) and the protocol provided. Triplicate wells were inoculated with 50,000 splenocytes/200 µl serum-free, X-VIVO 15 medium (Lonza, Walkerville, MD) and 10 µg/ml single peptide final concentration [100 mg/ml DMSO stock diluted to 1∶1,000] encoded by the vaccine constructs. Positive (phytohemagglutinin) and negative (0.1% DMSO) controls were included. The number of spots/well was quantified using a CTL-immunospot S5 UV Analyzer (Cellular Technology Limited, Shaker Heights, OH).

### Cytokine quantitation

Splenocytes (2×10^5^ cells)/200 µl X-VIVO 15 medium containing 10 µg/ml individual peptide/well in 96-well round bottom plates were incubated for ∼40 hours. The culture supernates were collected at the end of the incubation period; IL-4, IL-10, TNF-α and IFN-γ were quantified as we described previously using a MILLIPLEX MAP mouse cytokine/chemokine immunoassay kit (EMD Millipore Corporation; Billerica, MA) and the Bio-Plex 200 system (Bio-Rad Laboratories GmbH, Munich, Germany) in accordance with the manufacturers’ instructions [25].

### Statistical analysis

The results were analyzed using the SigmaStat statistics program (Systat Software, inc., San Jose, CA). Individual means were compared using a non-paired Student’s *t* test or a Mann-Whiney rank-sum test. Data derived from more than two groups were compared by one-way analysis of variance (ANOVA) followed by a Dunnett’s test to identify the groups that differed significantly from the control (*P*<0.05).

### Ethics Statement

Mice imported to Rhode Island Hospital were treated in accordance with NIH publications entitled “Principles for Use of Animals” and “Guide for the Care and Use of Laboratory Animals” (8^th^ Ed.) and an animal welfare protocol approved by Rhode Island Hospital’s Animal Care and Use Committee (Animal Welfare Protocol Committee number: 0256-11). Experimental animals were monitored visually on a daily basis for signs of distress that included hunching, ruffled fur, and impaired food and water intake. Pain-free euthanasia at the termination of a procedure was assured by carbon dioxide asphyxiation followed by cervical dislocation.

## Results

### CD11c^+^PDCA-1^+^ vector DCs

**Figure 2:**
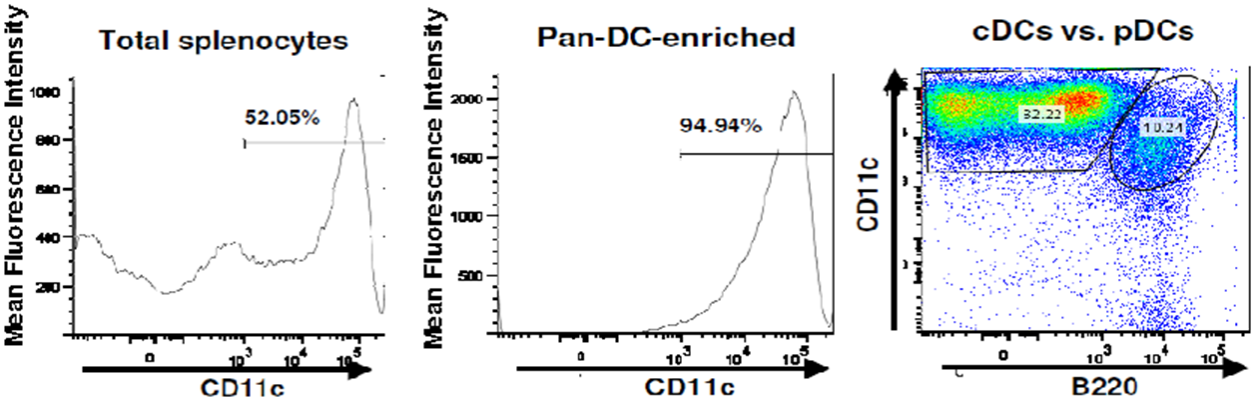
Purified CD11c^+^PDCA-1^+^ splenocytes. The total splenocyte population (left panel) derived from HLA-A2/DRB1 mice on day 12 post-inoculation with FLT3L-secreting melanoma cells was enriched positive selection using pan-DC magnetic MicroBeads (middle panel) and characterized by flow cytometry (right panel).

**Figure 3:**
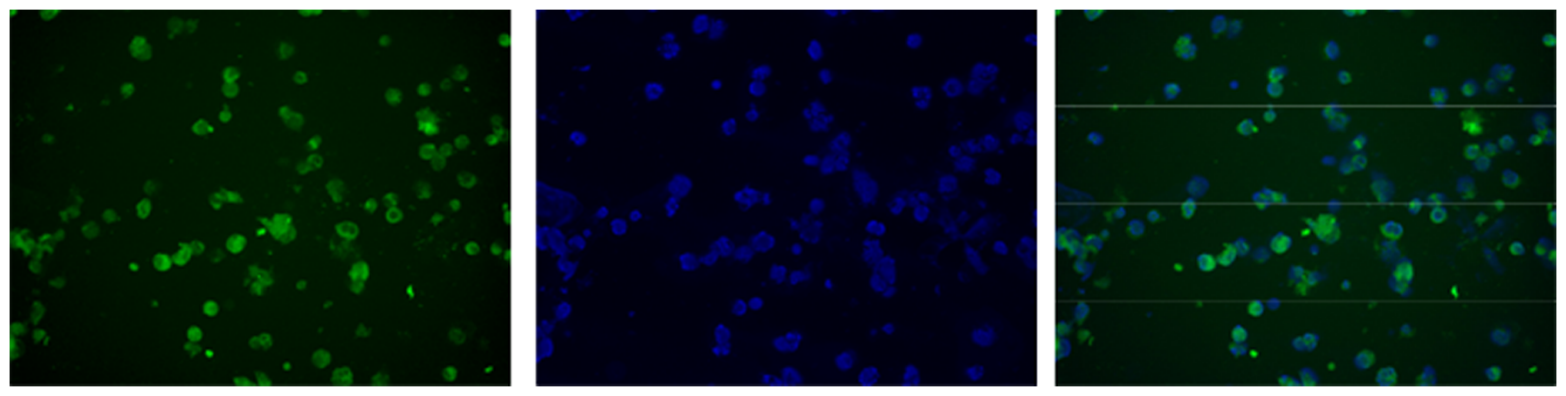
HLA-A2/DRB1 DCs transfected by electroporation. Purified HLA-A2/DRB1 DCs were transfection by electroporation with a GFP-expressing plasmid (NTC8685-eRNA41H-EGFP) and cultured overnight in the presence of GM-CSF to promote viability. The cells were examined visually on the following day: UV microscopy (left), DAPI-counter stain (center), merged images (right).

Humanized, HLA-A2/DRB1 transgenic mice inoculated s.c. with Flt3L-secreting B16 melanoma cells exhibited a 4- to 5-fold increase in spleen cell number and a dramatic expansion of the splenic DC population (Table 1 and Figure 2). The DC population, enriched to ≈95% purity by positive selection using pan-DC magnetic MicroBeads, was composed of conventional (c)DCs and plasmacytoid (p)DCs. Both subsets were relatively immature, evidenced by the minority of cells that expressed cell-surface HLA-class II (DR1), CD40 and CD80 molecules. [Notably, mice inoculated with Flt3L-secreting B16 melanoma cells also exhibited a pronounced increase in size of the hepatic DC population consisting of immature cDCs and pDCs (data not shown).] Subsequently, the purified splenic DC population was transfected by electroporation with NTC8685-eRNA41H-EGFP (GFP-expressing plasmid) in order to determine transfection efficiency. Microscopic examination of the cells on the following day revealed an approximate 60% transfection rate estimated visually (Figure 3).

**Table 1:** DCs derived from Flt3L-treated, HLA-A2/DRB1 mice exhibit an immature phenotype^a^.

Trait	CD11c^+^B220^−^	CD11c^+^B220^+^
	(conventional; cDCs)	(plasmacytoid; pDCs)
**DCs/spleen**	5.94×10^7^	0.65×10^7^
**CD8^+^**	62.30%	ND^b^
**CD11b^+^**	47.00%	ND
**CD40^+^**	0.90%	8.10%
**CD80^+^**	3.00%	15.10%
**CD86^+^**	51.30%	24.40%
**HLA-DR1^+^**	5.30%	6.40%

a CD11c^+^PDCA-1^+^ DCs, purified from 2.55×10^8^ splenocytes/HLA-A2/DRB1 transgenic mouse inoculated s.c. with Flt3L-secreting B16 myeloma cells 12 days previously, were quantified and characterized by flow cytometry.

b Not determined.

### DC vector-mediated vaccination against hepatitis C virus

**Figure 4:**
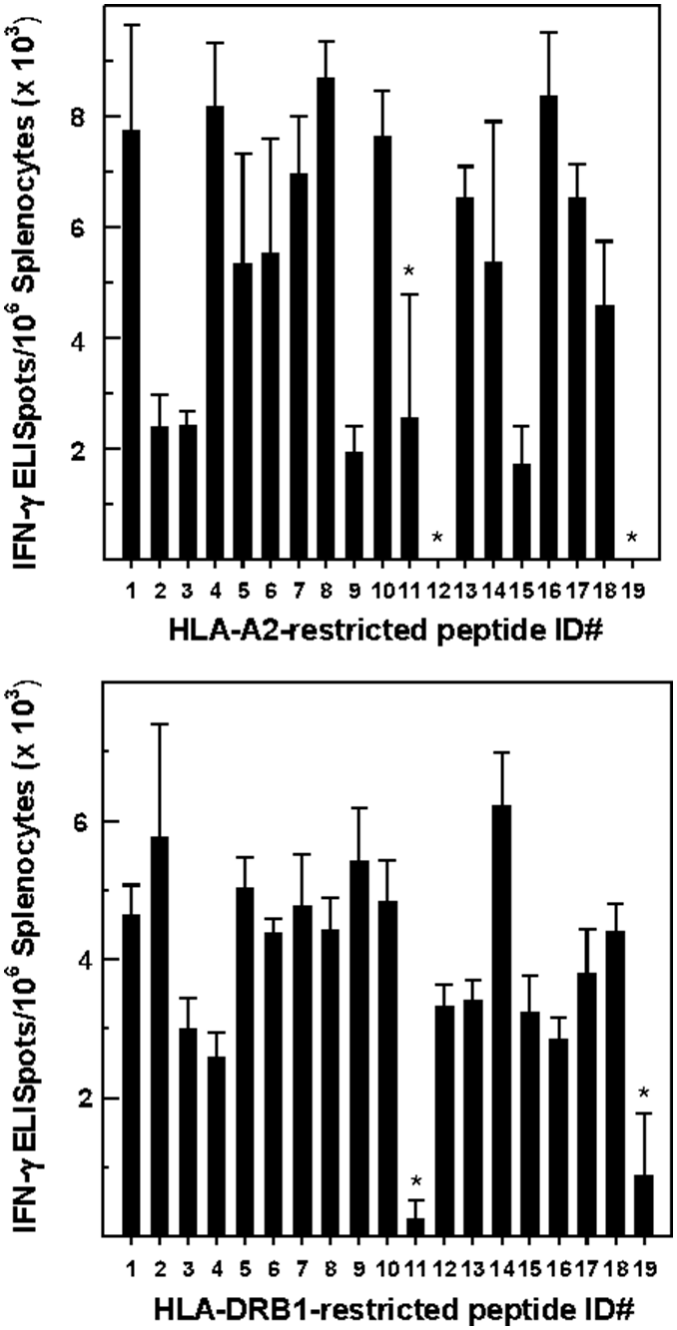
IFN-γ ELISpot assays. The spleens were dissected and pooled on day 35 from groups of 4 mice immunized with vaccine construct-transfected, and IFN-γ ELISpot assays were performed in triplicate. The data, expressed as the means ± SD ELISpots/10^6^ splenocytes minus the average negative control, 0.1% DMSO+2 SD (3,346 ELISpots/10^6^ splenocytes), were obtained in a single experiment representative of duplicate experiments. The average number of ELISpots/10^6^ splenocytes derived from mice immunized with non-transfected DCs was not significantly different from the DMSO control (not shown). *Splenocytes derived from mice immunized with transfected DCs and incubated with the HLA-A2- and -DRB1-restricted peptides indicated did not yield values that were significantly different from the DMSO control (ANOVA).

The gene sequences that encode highly conserved HLA-A*0201-restricted HCV epitopes and HLA-DRB1-restricted viral ICS (validated previously by their ability to elicit specific naïve human T cell responses *in* *vitro*) were incorporated into two separate multi-epitope genes, and each gene was cloned into an expression vector (NTC8685-eRNA41H-EGFP). HLA-A2/DRB1 transgenic mice were immunized with these vaccine constructs using DCs (characterized in the preceding section) as a vector. DCs transfected with either of the two constructs were mixed in equal proportion and the mice were immunized s.c. Mice administered non-transfected DCs served as the control. Splenocytes derived from a group of four mice on day 35 following immunization and a single boost 2-weeks later demonstrated a negligible response to most peptides encoded by the vaccine constructs in IFN-γ ELISpot assays (data not shown). Splenocytes derived from four mice vaccinated and boosted twice, however, recognized and produced IFN-γ in response to incubation with ∼90% of the HLA-A2-restricted epitopes and all the HLA-DRB1-restricted ICS (Figure 4). In both cases, the responses to individual peptides quantified in ELISpot assays varied in terms of the number of spots.

**Figure 5:**
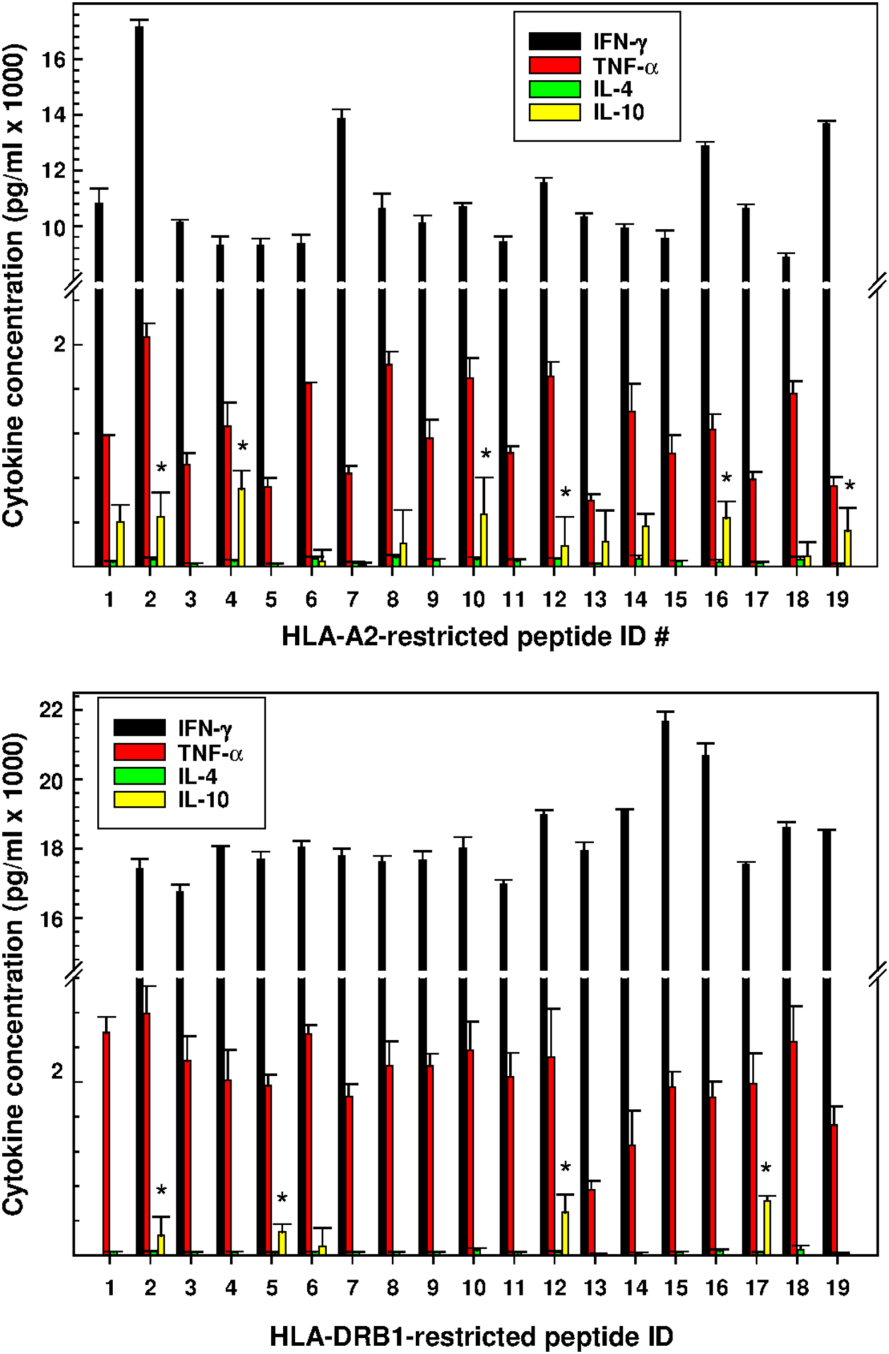
Cytokine bead array. The spleens were dissected and pooled on day 35 from groups of four mice immunized with vaccine construct-transfected or non-transfected DCs. Single cell suspensions were transferred to quadruplicate wells and cultured 40 hours in the presence of the peptide indicated. The data, obtained in a single experiment representative of two experiments, are the means ± SD pg/ml cytokine produced by splenocytes obtained from mice immunized with transfected DCs minus that produced by splenocytes derived from control mice immunized with non-transfected DCs. Concentrations of IFN-γ, TNF-α, IL-4 and IL-10 (where indicated by *) are significantly greater than that produced by splenocytes derived from control animals (*P<*0.05; Student’s *t* test or Mann-Whitney Rank Sums test).

Vaccination favored Th1-type cytokine production by splenocytes derived from vaccinated mice compared to mice inoculated with non-transfected DCs. Though the response to individual HLA-A2- and -DRB1-restricted peptides varied, the overall production of pro-inflammatory cytokines (TNF-α and IFN-γ) was elevated significantly relative to the production of IL-4 and IL-10 (Figure 5), Indeed, IL-4 production was barely detectable while IL-10 production was often not increased relative to the control.

## Discussion

New chemotherapeutic approaches that include NS3-4A protease and/or NS5B polymerase inhibitors administered with or without pegylated-interferon and ribavirin result in a significant increase in the SVR found in patients infected with HCV genotype 1 [7]. However, the adverse effects of therapy are often severe, the costs are high, and a significant portion of those patients treated remains infected [26]. Therapeutic vaccination alone or in combination with drug treatment offers an additional approach to treating chronic hepatitis C. Unfortunately, vaccination strategies undertaken to date have demonstrated varied and only limited success in clinical trials [9], [10]. Recombinant protein vaccines, for example, are safe and well tolerated, but generally ineffective due to their inability to induce CD8^+^ T cell responses. Peptide-based HCV vaccines are similarly ineffective despite their ability to elicit weak, epitope-specific CD8^+^ T cell responses and a transient reduction in viral load in a minority of chronically infected patients. Likewise, four out of six patients vaccinated with a DNA construct that encoded conserved nonstructural (NS)3 and NS4A peptide sequences exhibited an extremely modest reduction in serum viral load [27]. Finally, attenuated viral vectors (e.g., adenovirus or modified vaccinia virus Ankara [MVA]) have been used to boost the epitope-specific responses of CD4^+^ and CD8^+^ T cells. Recombinant adenovirus that encoded the NS3–NS5 region of HCV genotype 1B, for example, induced the sustained, multiple epitope-specific T cell response of healthy volunteers [28]. However, only 8 of 15 chronically infected patients vaccinated with MVA engineered to express HCV NS3-5b proteins exhibited a reduction in viral load, and this reduction was transient [29].

Immunotherapy with HCV epitope-expressing DCs offers an alternate, vector-mediated approach to treating chronic HCV-infected patients; the results of a recent clinical trial document the potential effectivity [12], [13]. Vaccination induced the response of CD8 T cells specific for six HLA-A2 restricted peptides expressed by the DC vector; the response was transient, however, and the effect on viral load was minimal. The goal of the present study was to provide additional support for this approach, envisioning that an increased range of HLA-A2-restricted epitopes and including HLA-DRB1-restricted epitopes expressed by the vector would improve the clinical outcome of vaccination. For this purpose, the gene sequences encoding 38 highly conserved HCV epitopes/ICS (identified previously and listed in Tables S1 and S2 in File S1) were incorporated into two vaccine constructs that encoded either HLA-A2- or –DRB1-restricted epitopes [14]. Subsequently, immature DCs derived from humanized, HLA-A2/DRB1 (donor) mice were transfected with these two constructs and the transfected DCs were used to immunize HLA-A2/DRB1 (recipient) mice. The vast majority of the epitopes encoded by these constructs induced peptide-specific T cell recognition and the production of TNF-α and IFN-γ by immune splenocytes (Figure 5). Both cytokines are principal mediators of anti-HCV-specific T cell responses [30], [31]. High-avidity, HLA-A2-restricted T cells recovered from patients who cleared the virus exhibited enhanced IFN-γ and TNF-α production [31]. Moreover, CD4^+^ and CD8^+^ T cells obtained from healthy volunteers vaccinated with two recombinant adenoviral vectors that encoded the NS3–NS5 region of HCV genotype 1B secreted IFN-γ and TNF*-*α [28].

In contrast to TNF-α and IFN-γ, the epitope-specific production of T_H2_-type cytokines (i.e., IL-4 and IL-10) by splenocytes derived from vaccinated HLA-A2/DRB1 transgenic mice was often non- or barely detectable. It is relevant to note in this regard, that IL-10 typifies the progression to chronic liver disease in patients infected with HCV [32]. Moreover, while IFN-γ and TNF-α production is elevated, the production of IL-4 and IL-10 by T cells derived from patients who achieved a SVR to chemotherapy is significantly diminished [33], [34].

The response of regulatory T cells to certain epitopes derived from structural, as well as non-structural, HCV proteins including core, NS3 and NS5 is well documented [35]–[37]. Thus, peptide- or epitope-based vaccine strategies that exclude such epitopes offer a significant advantage over strategies that utilize intact HCV proteins or vectors that encode whole proteins, which include regulatory T cell epitopes and, consequently, can impede rather than promote viral clearance. Interestingly, one HLA-DRB1-restricted hepatitis C ICS (identified initially during screening, but not included in the vaccine construct described herein) exhibited extensive human homology and the ability to elicit the response of regulatory T cells within the PBMC population derived from HCV infected, but not non-infected, individuals (Losikoff *et al.,* manuscript submitted). Furthermore, vaccination with DNA constructs that express multiple viral epitopes, rather than the epitopes/peptides themselves, circumvents any uncertainty regarding the longevity of epitope presentation [38].

Previously, we reported that human monocyte-derived DCs pulsed with the same HLA-A2- and –DRB1-restricted peptide sequences encoded by the vaccine constructs described herein induced epitope-specific TNF-α and IFN-γ production by naïve CD4^+^ and CD8^+^ human T cells *ex vivio* [14]. These results, taken in conjunction with those reported here, support the therapeutic potential of DCs expressing a broad array of HLA-A2- and –DRB1-restricted epitopes in treating chronic, HCV-infected patients. Clearly, however, any effort to demonstrate the efficacy of a therapeutic vaccine against chronic hepatitis C is hampered by the lack of a good, immunocompetent rodent model that supports viral infection and replication [39]. Moreover, while immunization with DCs that express an increased number of HLA class I- and class II-restricted epitopes offers to improve vaccine efficacy relative to that reported previously [12], [13], such *ex* *vivo* therapies suffer from a number of practical limitations including the estimated high cost of treatment and a failure to elicit innate immunity. As an alternative approach, we envision therapeutic vaccination with a multiple HCV epitope-expressing DNA vector, e.g., attenuated *Listeria monocytogenes*, that targets DCs *in* *situ* and stimulates both innate and adaptive immunity [40]. Such a vector-mediated, epitope-based vaccine offers an additional approach to treating the expanding patient population affected by chronic hepatitis C, as well as immunizing a non-infected population at risk.

## Supporting Information

File S1 **This includes Tables S1 and S2.**
(DOC)
