# Supplementary material for: Dendritic Cell-Mediated, DNA-Based Vaccination against Hepatitis C Induces the Multi-Epitope-Specific Response of Humanized, HLA Transgenic Mice
Source: PLoS One. 2014 Aug 11;9(8):e104606. doi: 10.1371/journal.pone.0104606 (PMC4128787; doi:10.1371/journal.pone.0104606)
Supplement: File S1 — This includes Tables S1 and S2. (DOC) [file pone.0104606.s001.doc]

**Supporting Information**

**Table SI.** HLA-A*0201-restricted HCV epitopes

| Peptide ID# | Name | Sequence |
| --- | --- | --- |
| **1** | HCV_G1_NS4b_1917 | WMNRLIAFA |
| **2** | **HCV_G1a_NS5b_2734** | MLVCGDDLV |
| **3** | **HCV_G1_NS4b_1765** | HMWNFISGI |
| **4** | **HCV_G1_NS3_1451** | SVIDCNTCV |
| **5** | **HCV_G1b_NS5b_2829** | WLGNIIMYA |
| **6** | **HCV_G1_E2_615** | RLWHYPCTV |
| **7** | **HCV_NS3_1586** | YLVAYQATV |
| **8** | **HCV_G1_E2_685** | ALSTGLIHL |
| **9** | **HCV_G1_core_133** | DLMGYIPLV |
| **10** | **HCV_G1_NS3_1074** | CINGVCWTV |
| **11** | **HCV_G1_E1_323** | MMMNWSPTT |
| **12** | **HCV_G1_E1_364** | SMVGNWAKV |
| **13** | **HCV_G1_NS3_1274** | GIDPNIRTGV |
| **14** | **HCV_G1_NS4b_1808** | LLFNILGGWV |
| **15** | **HCV_G1_NS3_1607** | QMWKCLIRL |
| **16** | **HCV_G1_NS5b_2559** | IMAKNEVFCV |
| **17** | **HCV_G1_E1_277** | YVGDLCGSV |
| **18** | **HCV_G1_E1_281** | DLCGSVFLV |
| **19** | **HCV_G1_NS5b_2945** | YLFNWAVRT |

**Table SII.** HLA-DRB1-restricted HCV ICS

| Peptide ID# | Name | Sequence |
| --- | --- | --- |
| **1** | **HCV_G1_NS3_1246** | AQGYKVLVLNPSVAATLGFG |
| **2** | **HCV_G1_NS4b_1876** | VDLLVNLLPAILSPGA |
| **3** | **HCV_G1_NS5b_2879** | LGNIIQRLHGLSAFSLHSY |
| **4** | **HCV_G1_NS4b_1769** | ISGIQYLAGLSTLPGNPA |
| **5** | **HCV_G1_NS4b_1941** | AARVTQILSSLTITQLLKRLHQWI |
| **6** | **HCV_G1_NS5b_2440** | KLPINALSNSLLRHH |
| **7** | **HCV_G1_NS4b_1725** | AEQFKQKALGLLQTASRQAE |
| **8** | **HCV_G1a_NS5b_2485** | LQVLKEVKAAASKVKANL |
| **9** | **HCV_G1_NS4b_1790** | LMAFTAAVTSPLTTS |
| **10** | **HCV_G1_NS5b_2840** | WARMILMTHFFSVLIARDQLEQ |
| **11** | **HCV_G1_E2_732** | AYCLWMMLLISQAEAALELIT |
| **12** | **HCV_G1a_E1_255** | AAILRRHIDLLVGSATLCSALY |
| **13** | **HCV_G1_NS3_1605** | DQMWKCLIRLKPTLHGPTP |
| **14** | **HCV_G1_NS5b_2941** | CGKYLFNWAVRTKLKLT |
| **15** | **HCV_G1a_E1_359** | GIAYFSMVGNWAKVL |
| **16** | **HCV_G1a_NS2_909** | VPYFVRVQGLLRICALARKAV |
| **17** | **HCV_G1b_NS5b_2898** | PGEINRVASCLRKLGVPPLRAY |
| **18** | **HCV_G1b_NS5b_2913** | VPPLRVWRHRARSVRAKLLSQGGRA |
| **19** | **HCV_G1b_NS2_748** | LENLVVLNAASVAGAHW |
